# Synergistic Remediation of Organic Dye by Titanium Dioxide/Reduced Graphene Oxide Nanocomposite

**DOI:** 10.3390/molecules28217326

**Published:** 2023-10-29

**Authors:** Martina Kocijan, Lidija Ćurković, Damjan Vengust, Tina Radošević, Vasyl Shvalya, Gil Gonçalves, Matejka Podlogar

**Affiliations:** 1 Department of Materials, Faculty of Mechanical Engineering and Naval Architecture, University of Zagreb, Ivana Lučića 5, 10000 Zagreb, Croatia; 2 Advanced Materials Department, Jožef Stefan Institute, Jamova Cesta 39, SI-1000 Ljubljana, Slovenia; damjan.vengust@ijs.si; 3 Department for Nanostructured Materials, Jožef Stefan Institute, Jamova Cesta 39, SI-1000 Ljubljana, Slovenia; tina.radosevic@ijs.si (T.R.); matejka.podlogar@ijs.si (M.P.); 4 Department of Gaseous Electronics, Jožef Stefan Institute, SI-1000 Ljubljana, Slovenia; vasyl.shvalya@ijs.si; 5 Centre for Mechanical Technology and Automation (TEMA), Mechanical Engineering Department, University of Aveiro, 3810-193 Aveiro, Portugal; ggoncalves@ua.pt; 6 Intelligent Systems Associate Laboratory (LASI), 4800-058 Guimarães, Portugal

**Keywords:** photocatalysis, TiO_2_@rGO, methylene blue, water environments

## Abstract

In this work, nanocomposites based on titanium dioxide and reduced graphene oxide (TiO_2_@rGO) with different weight percentages of rGO (4, 8, and 16 wt%) were prepared by the hydrothermal/solvothermal synthesis method and thermally treated at 300 °C. The prepared nanocomposites were explored for the removal of methylene blue dye (MB) in the presence of simulated solar illumination as well as natural sunlight. The structural, morphological, chemical, and optical properties of the as-synthesized TiO_2_@rGO nanocomposites were characterized. The obtained results of the graphene-based nanocomposite materials indicated the existence of interactions between TiO_2_ and rGO, i.e., the Ti–O–C bond, which confirmed the successful integration of both components to form the TiO_2_@rGO nanocomposites. The addition of rGO increased the specific surface area, decreased the band gap energy, and increased the photocatalytic degradation efficiency of MB from water compared to TiO_2_ nanoparticles. The results of photocatalytic activity indicated that the amount of rGO in the prepared TiO_2_@rGO nanocomposites played a significant role in the application of different photocatalytic parameters, including the initial dye concentration, catalyst concentration, water environment, and illumination source. Our studies show that the reinforcement of the nanocomposite with 8 wt% of rGO allowed us to obtain the maximum photocatalytic decomposition performance of MB (10 mg·L^−1^) with a removal percentage of 99.20 after 2 h. Additionally, the obtained results show that the prepared TiO_2_@rGO_8 wt% nanocomposite can be used in three consecutive cycles while maintaining photocatalytic activity over 90%.

## 1. Introduction

Water is an essential element for life on Earth, regulating the health and prosperity of the ecosystem. However, water quality is becoming increasingly degraded due to pollution with various organic and inorganic compounds, resulting from uncontrolled population growth worldwide and industrialization [1]. Wastewater leads to the serious pollution of surface water, underground water, and soil. Even a very small concentration of pollutants (less than 1 mg·L^−1^) has a strong negative impact on the whole ecosystem. Furthermore, the concentrations of pollutants in the environment will depend on the toxicity and/or biodegradability of the contaminants. The World Bank estimates that roughly a fifth of the global polluted industrial water comes from wastewater treatment and textile dyeing. Wastewater from dyeing contains high concentrations of dangerous compounds, detergents, oils, soaps, fats, sulfides, soda, and waste-containing alkalis [2]. Wastewater-containing dyes are discharged into rivers, seas, or oceans and are not biodegradable due to their stability and complex aromatic structure (azo, anthraquinone, sulfur, triphenylmethyl, and phthalocyanine groups), and this inhibits the penetration of light into the depths of the aquatic environment. As a result, there are serious ecological consequences, such as changes in the quality of the aquatic environment, e.g., the color and smell of the water, as well as toxicity. Moreover, it causes the inhibition of sunlight diffusion, which is necessary for the process of photosynthesis in aquatic flora and fauna. It also prevents the oxygen transfer mechanism, an important parameter of aquatic life at the air–water interface [3,4].

Advanced oxidation processes attract attention due to the possibility for the complete removal of many pollutants through oxidation and reduction reactions in water. Among them, semiconductor-based photocatalysis stands out as an effective process in wastewater treatment, where the reaction process is supported by photons in the presence of a semiconductor catalyst [5]. The main role in advanced oxidation processes is played by the hydroxyl radical (^•^OH) due to its high reactivity and strong oxidation potential, which is *E*° = 2.8 V [6,7]. Using advanced oxidation processes, it is expected that the hydroxyl radical, as a strong oxidizing agent, may completely decompose and mineralize organic pollutants from wastewater into non-toxic compounds such as water, carbon dioxide, and related mineral acids [8,9]. Advanced oxidation processes prove to be efficient and more environmentally friendly for the removal of persistent water pollutants such as dyes, pesticides, pharmaceuticals, etc. [10,11,12,13].

Titanium dioxide (TiO_2_) is an *n*-type semiconductor material with a wide band gap energy of 3.2 eV for the anatase phase [14]. After the splitting of water into hydrogen and oxygen using TiO_2_ by Fujishima and Honda in 1972, TiO_2_ gained attention in the field of science and technology [15]. Its exceptional properties, such as thermal, mechanical, and chemical stability, non-toxicity, and a low cost, make it the basis of application in many fields. The practical application of TiO_2_ is very widespread, including additives in paints, plastics, and personal care products such as cosmetics and sunscreens [16]. It is also used in medicine, energy storage, and air and water purification. Its role in catalytic applications is especially highlighted since TiO_2_ shows excellent photocatalytic activity in many processes involved in removing pollutants from the environment using UV illumination. The main disadvantage of TiO_2_ is the high band gap energy and fast recombination of electrons and holes [17]. A strategy to overcome the limitations of TiO_2_ photocatalysts is based on nanocomposite materials, with the aim of developing photocatalytic materials with a lower band gap energy and reduced electron–hole recombination (*e*^−^–*h*^+^). The modification of TiO_2_ with carbon-based materials such as graphene or its derivatives (graphene oxide (GO) and reduced graphene oxide (rGO)) is very promising due to their large specific surface area. Moreover, graphene derivatives play a major role in photocatalytic processes because they are excellent compounds for electron transfer. The excellent conductivity of rGO has the potential to separate photogenerated *e*^−^–*h*^+^ pairs and reduce recombination [18]. In the literature, it has been shown that a TiO_2_@rGO nanocomposite has improved photocatalytic properties compared to pure TiO_2_ [19,20,21,22].

Several studies using TiO_2_@rGO nanocomposites have been conducted to degrade organic pollutants from water. Duan et al. [23] treated 4-nitrophenol using a TiO_2_@rGO nanocomposite for 6 h, exposing it to simulated sunlight (Xenon lamp, 300 W) illumination, and reported complete photodegradation. In another study by Deshmukh et al. [22], the photocatalytic degradation of MB using a TiO_2_@rGO nanocomposite was 91.3% at the end of 30 min for pH 13.2 and a photocatalyst dosage of 2 g·L^−1^ under natural sunlight illumination. Furthermore, Kusiak-Nejman et al. [19] studied the photocatalytic degradation of MB while using the same rGO@TiO_2_ nanocomposites under UV irradiation (Philips, 20 W). They reported high effectiveness, degrading 91.48% of MB of organic content under 1 h of illumination. The prepared TiO_2_@rGO nanocomposite showed long-term stability after seven cycles (decreased from 100% to 73.3% after 2 h). Wu et al. [24] studied the photocatalytic degradation of Rhodamine B (RhB) and reported ~95% removal within 90 min of UV irradiation (250 W high-pressure Hg lamp) at room temperature. The authors also studied the TiO_2_@rGO nanocomposite’s reusability; the results obtained after five cycles indicated that the prepared nanocomposite was rather stable, with over 90% removal capabilities.

The main objective of this scientific research was to develop a TiO_2_@rGO nanocomposite for the efficient degradation of MB from water under simulated solar illumination emulating environmental conditions. Nanocomposites based on two components, titanium dioxide and reduced graphene oxide (TiO_2_@rGO), with different weight percentages of rGO of 4, 8, and 16 wt%, were prepared by the hydrothermal/solvothermal synthesis method and afterwards thermally treated at 300 °C. The synthesized photocatalysts were characterized by different structural, morphological, chemical, and optical characterization methods. The adsorption and photocatalytic activity of the prepared TiO_2_@rGO nanocomposites was tested in terms of the efficiency of removal of MB dye from water. Furthermore, the photocatalysts’ performance was optimized by changing various parameters, such as the irradiation source, concentration of catalyst, initial concentration of the dye model solution, and water matrix. The recyclability of the TiO_2_@rGO photocatalyst under optimized conditions was also assessed.

## 2. Results and Discussion

### 2.1. Characterization of Materials

**Figure 1:**
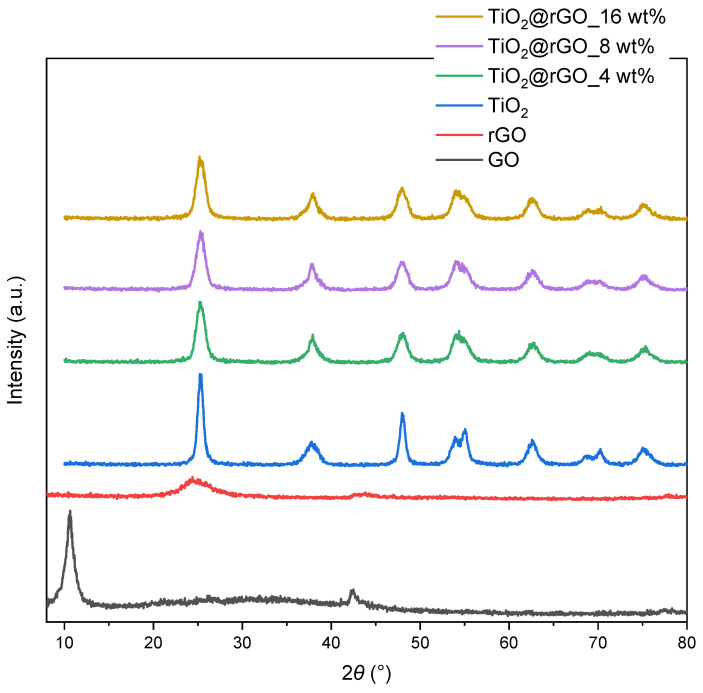
Diffractograms of samples of GO, rGO, TiO_2_ nanoparticles, and TiO_2_@rGO nanocomposites prepared with different additions of rGO (4, 8, and 16 wt%).

The structural analysis of the TiO_2_@rGO nanocomposite materials was first performed by XRD analysis. The crystalline phase of the prepared GO, rGO, TiO_2_ nanoparticles, and TiO_2_@rGO nanocomposites was determined by X-ray powder diffraction analysis (Figure 1). The diffractogram of GO showed two diffraction maxima at 2θ = 10.61° and 2θ = 42.45°. According to the literature, the oxidation of graphite to form GO leads to an increase in the carbon interplanar distance, forming a characteristic peak at 2θ = 10.61°. On the other hand, the reduction of GO promotes the formation of a broadening peak at 2θ = 24.5°, resulting from the decreasing of the interplanar distance between the carbon planes, which is due to the elimination of the oxygen functional groups [25,26,27].

The diffraction maximum of weak intensity appear at 2θ = 42.45°, which is attributed to the crystal plane (100) of the honeycomb hexagonal structure of graphite. The diffractogram of the rGO sample shows a broad diffraction maximum at 2θ = 24.48°, which is a consequence of the reduction of GO by removing surface oxygen functional groups [28]. The 2θ diffraction maxima of TiO_2_ occur at 25.30°, 37.83°, 48.02°, 53.90°, 55.05°, 62.61°, 68.72°, 70.27°, and 75.21°, corresponding to the crystal planes (101), (004), (200), (105), (211), (204), (116), (220), and (215) of the anatase phase (JCPDS No. 21-1272) [29,30]. With the addition of different amounts of rGO, we observed that the diffraction maxima of the anatase phase of TiO_2_ were still present. The intensity of the diffraction maximum of the (101) plane in TiO_2_@rGO nanocomposites was reduced and became wider compared to pure TiO_2_. As a result, the diffraction maximum of the (101) plane overlapped with the diffraction maximum of the (002) plane of rGO. However, neither the presence of other phases nor a significant shift in the diffraction maxima was observed with the addition of different amounts of rGO.

**Figure 2:**
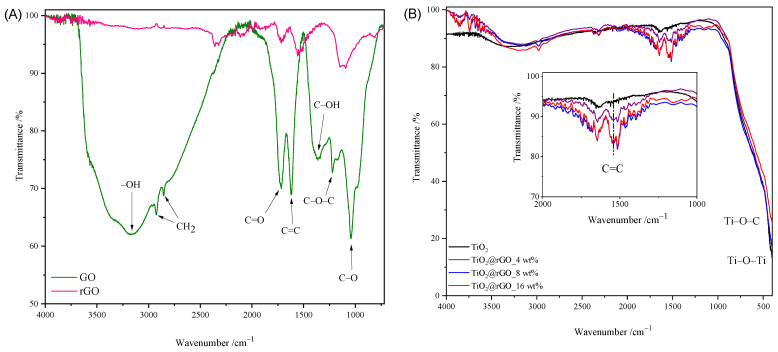
FTIR spectrum of (**A**) GO and rGO, (**B**) TiO_2_ nanoparticles, and TiO_2_@rGO nanocomposites.

The FTIR spectra of GO, rGO, TiO_2_ nanoparticles, and TiO_2_@rGO nanocomposites are shown in Figure 2. The broad peak between 3500 cm^−1^ and 2500 cm^−1^ in the FTIR spectrum of GO is due to carboxylic O–H stretching. The absorption peak at 3372 cm^−1^ corresponds to the stretching of the OH group of the carboxylic acid, which is a consequence of the presence of absorbed water molecules and alcohol groups [31]. The peaks corresponding to absorption at 2925 cm^−1^ and 2857 cm^−1^ are due to the asymmetric and symmetric CH_2_ stretching of graphene oxide [32]. The peak at 1715 cm^−1^ is attributed to the C=O stretching of the carboxyl and carbonyl groups. The absorption at 1622 cm^−1^ is attributed to sp^2^-hybridized C=C stretches from the unoxidized graphitic structure of the aromatic ring [33]. The deformational stretching vibration of the C–OH hydroxyl group is shown at 1376 cm^−1^ [34]. The vibration at 1221 cm^−1^ is attributed to the stretching of the C–O–C epoxy group, while the vibration at 1039 cm^−1^ is attributed to the stretching of the C–O (alkoxy group) bond in the epoxy group (C–O–C) [33,35]. From the comparison of the spectra of GO and rGO shown in Figure 2A, it is visible that the intensities of the absorption values attributed to the oxygen functional groups are significantly reduced or completely disappear. The obtained spectrum of rGO confirmed the successful hydrothermal–thermal reduction of rGO.

The FTIR spectra of the prepared TiO_2_ and TiO_2_@rGO nanocomposites with different proportions of rGO are shown in Figure 2B. Compared to the spectrum of graphene oxide, a small number of oxygen functional groups appear on the surfaces of the prepared TiO_2_@rGO nanocomposites. In the FTIR spectra of the prepared TiO_2_@rGO nanocomposites, a wide vibration band appears in the range from 400 cm^−1^ to 900 cm^−1^, which is attributed to the stretching of the Ti–O–C bond, while, for TiO_2_ nanoparticles, the absorption band corresponds to the Ti–O–Ti vibration [36,37]. The C=C bond was identified by the FTIR analysis of the prepared nanocomposites.

**Figure 3:**
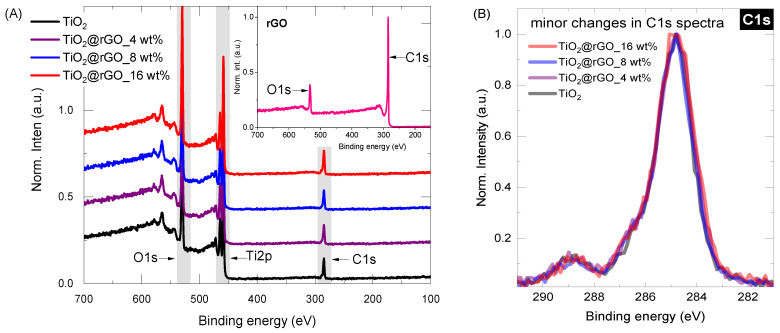
(**A**) XPS survey spectra for the selected Ti-containing samples (inset represents XPS of rGO) and (**B**) carbon C1S core level spectra for Ti-containing samples.

Surface chemistry was investigated for all samples (rGO, TiO_2_, TiO_2_@rGO_4 wt%, TiO_2_@rGO_8 wt%, TiO_2_@rGO_16 wt%) by conducting long-range surveys and obtaining high-resolution spectra of the core elements (C1s, O1s, Ti2p). From the survey data (Figure 3A), it is possible to observe that the carbon content increases slightly with the addition of rGO, from 19.9% in the pure TiO_2_ (high carbon content associated with precursor used, Ti(C_3_H_5_O_12_)_4_) to about 24.5% in the TiO_2_@rGO_16 wt% mixture. The increasing trend in the carbon share, as shown in Table 1, varies according to the nominal concentration of rGO in the TiO_2_@rGO mixture. Even after normalizing the carbon C1s spectra for the Ti-containing samples, there is no significant change in the curve profile (Figure 3B). The concentration analysis of each relevant element (C, O and Ti) can be found in Table 1.

In the case of rGO, the XPS shape of both curves (Figure S1, C1s, and O1s) resembles those typically observed in the literature [38,39,40]. The C1s peak was deconvoluted into five major components: (a) C=C, graphitic carbon with sp^2^ hybridization at ~284.7 eV; (b) the carbon in C–O at ~285.5 eV; (c) the epoxy/ether group (C–O–C, ~286.5 eV); (d) the carbonyl carbon (C=O, ~288 eV); and (e) the carboxylate carbon (H–O–C=O, ~289 eV). The deconvolution of the O1s peak further confirms the analytical information of the C1s components. The O1s spectrum can be generalized by the chemical components at ~531 eV and ~533 eV, which were assigned to the contributions of the C=O and C–O groups, respectively.

**Figure 4:**
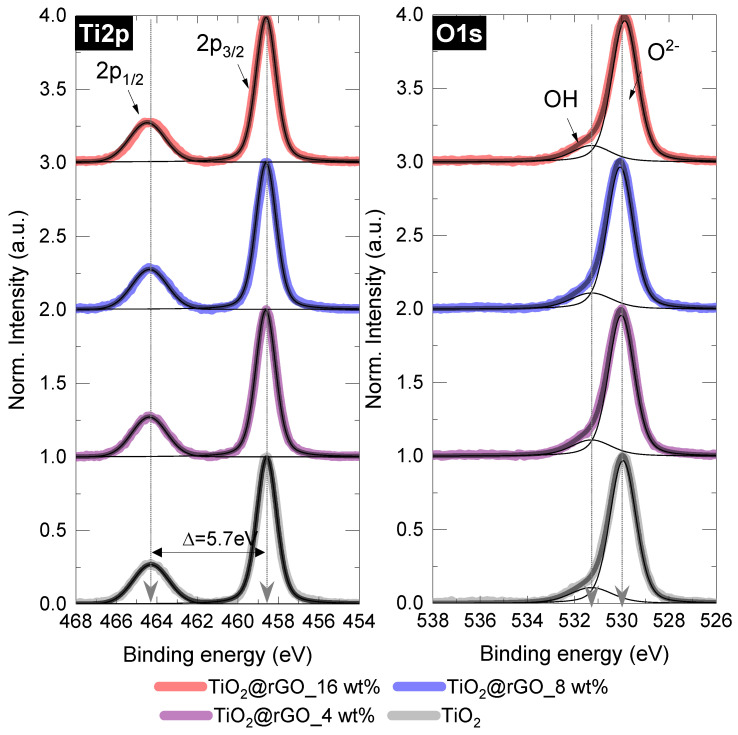
Deconvoluted Ti2p and O1s XPS spectra of the TiO_2_@rGO samples with different rGO content.

After normalization and background corrections, the high-resolution spectra of the mixed samples were deconvoluted and compared with those of pure TiO_2_ (Figure 4 and Table S1). In the case of the Ti2p peak, no significant changes were found, as all important parameters (FWHM of the splitting, position, peak area ratio) matched those of the pure TiO_2_ sample. Similarly, for the O1s components, the dominant lattice component (Ti–O or O^2−^) at 531.3 eV and the small OH peak showed negligible changes when TiO_2_ was mixed with rGO under the conditions used [41,42].

**Figure 5:**
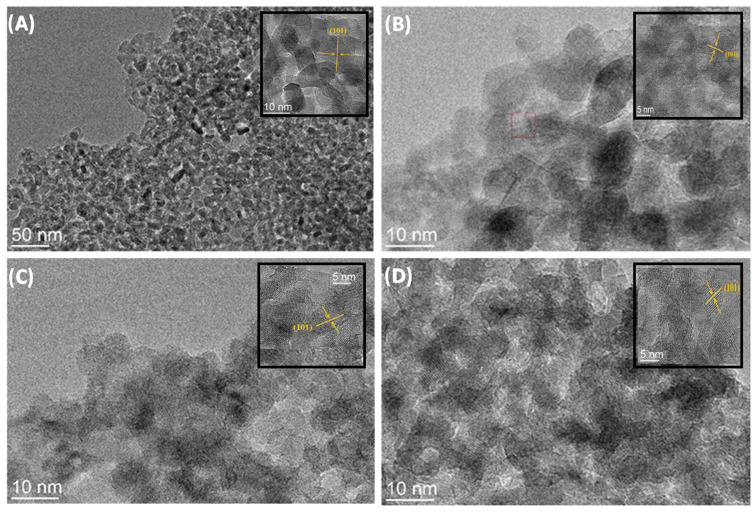
TEM micrographs of (**A**) TiO_2_ nanoparticles and nanocomposites: (**B**) TiO_2_@rGO_4 wt%, (**C**) TiO_2_@rGO_8 wt%, and (**D**) TiO_2_@rGO_16 wt%.

Figure 5A–D show TEM micrographs of samples of TiO_2_ nanoparticles and TiO_2_@rGO nanocomposites. The TEM micrographs show that all prepared samples consisted of nanocrystalline agglomerates of spherical morphology. The micrographs show the wrapping of the TiO_2_ nanoparticles around the rGO sheets. The interplane distance obtained by micrograph analysis is 0.18 nm, which corresponds to the (101) plane of the anatase phase of the TiO_2_ crystal structure.

**Figure 6:**
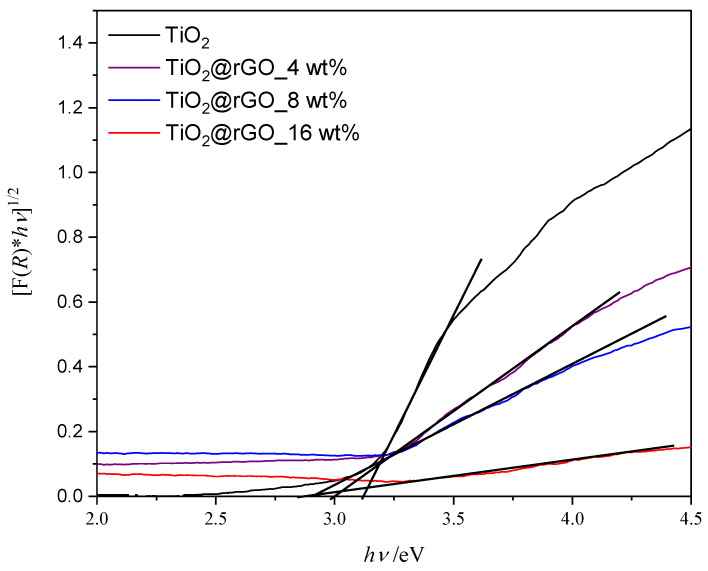
Tauc’s graphic for the indirect electron transition of prepared samples of TiO_2_ nanoparticles and TiO_2_@rGO nanocomposites.

The band gap energy of the prepared materials is shown graphically in Figure 6. The band gap energy values for photocatalysts TiO_2_, TiO_2_@rGO_4 wt%, TiO_2_@rGO_8 wt%, and TiO_2_@rGO_16 wt% are 3.12, 3.00, 2.91, and 2.89 eV, respectively. The results show that increasing the amount of rGO narrows the energy band in the prepared TiO_2_@rGO nanocomposites. The specific surface area of TiO_2_ nanoparticles and the TiO_2_@rGO_8 wt% nanocomposite is 134 and 168 m^2^·g^−1^, respectively. The integration of GO with a high specific surface area improves the overall specific surface area of the TiO_2_ nanocomposite [43]. Although the establishment of the relation between the specific surface area and adsorption and photodegradation mechanisms is not straightforward, it is possible to postulate some correlations. On one hand, adsorption is mainly governed by the chemical interactions between the GO aromatic structure and the MB molecule. On the other hand, the photocatalytic activity of the nanocomposite is dependent on the density of TiO_2_ active sites able to promote the photodegradation of MB. It is expected that an improvement in the specific surface will correspond to an increase in adsorption and photodegradation activity. Nevertheless, the photodegradation performance also depends on the change in the band gap energy of the nanocomposite when compared to pure TiO_2_ [44].

### 2.2. Photocatalytic Performance

Figure S2A shows the spectra of the photocatalytic degradation of MB with the use of the TiO_2_ photocatalyst, while Figure S2B–D show the decomposition with the use of nanocomposites TiO_2_@rGO_4 wt%, TiO_2_@rGO_8 wt%, and TiO_2_@rGO_16 wt%, respectively. During illumination with simulated solar radiation (specifications of the bulb are given in Table 2), all absorbance peaks decreased, which pointed to the decomposition of the MB dye structure, i.e., the removal of aromatic structures and auxochromic groups [45]. Based on the obtained results, the reduction in aromaticity and the decolorization of MB using the prepared photocatalysts is significant. The UV–Vis spectrum of MB has two main peaks, one at 664 nm due to the substitution of >N(CH_3_)_2_ groups on the heteroaromatic ring, which is responsible for the color of MB, and the other at 292 nm that is associated with the localized bands of the unsaturated heteroaromatic system [46,47,48]. During the decomposition of MB with the application of simulated solar radiation, a blue shift can be observed, i.e., a shift in the main absorption peak from 664 nm towards shorter wavelengths, i.e., to 645 nm for TiO_2_ and TiO_2_@rGO_4 wt%, at 628 nm for TiO_2_@rGO_8 wt%, and at 632 nm for TiO_2_@rGO_16 wt% [49]. The blue shift is attributed to the formation of N-demethylated intermediates [50,51]. The intensity of the peak at 664 nm was significantly reduced for all prepared photocatalysts after 120 min of reaction. It completely disappeared with the use of the TiO_2_@rGO_8wt% nanocomposite, which can be explained by the fact that not only the demethylation or decolorization of the solution occurred, but also the removal of the aromatic structure of MB. The intensity of the peak at 292 nm was also significantly reduced and showed a smaller blue shift at 288 nm for TiO_2_ nanoparticles and the TiO_2_@rGO_4 wt% nanocomposite and at 287 nm for the TiO_2_@rGO_8 wt% and TiO_2_@rGO_16 wt% nanocomposites. The blue shift in the peak at 292 nm confirmed the removal of the aromatic structure of MB with decolorization [50,52].

The largest decrease in the relative absorbance of the MB dye was achieved using the TiO_2_@rGO_8 wt% nanocomposite, as shown in Figure S3. The pseudo first-order rate constants and changes in the relative absorbance of the methylene blue peak at 664 nm were slightly faster compared to the pseudo first-order rate constants and changes in the relative absorbance of the MB peak at 292 nm (Table S2). Based on the obtained results of the MB degradation efficiency from the aqueous medium for both characteristic peaks at 292 and 664 nm using the prepared photocatalysts under simulated solar radiation, it was decided that the efficiency of removal would be monitored for *λ*_max_ = 664 nm.

#### 2.2.1. Effect of Photocatalyst Concentration

The amount of catalyst is one of the main factors affecting the efficiency of pollutant degradation in the photocatalytic process. To avoid the use of an excessive amount of catalyst, which can cause the agglomeration of catalyst particles, and to confirm the maximum absorption of light photons for the efficient removal of pollutants, the use of an optimal amount of catalyst is extremely important in the photocatalytic process [53]. Although there are many reports of conducted research related to the amount of catalyst used in the photocatalytic process, a direct comparison between studies is not possible due to the different parameters of the photocatalytic process, such as radiation flows, selected wavelengths, and the dimensions of the reactor itself. Here, the optimal amount of catalyst for the photocatalytic decomposition of MB under simulated solar radiation and using TiO_2_ nanoparticles and the TiO_2_@rGO nanocomposite (with a different mass weight of rGO) was tested for three different mass concentrations of the catalyst (0.1, 0.5, and 1 g·L^−1^).

**Figure 7:**
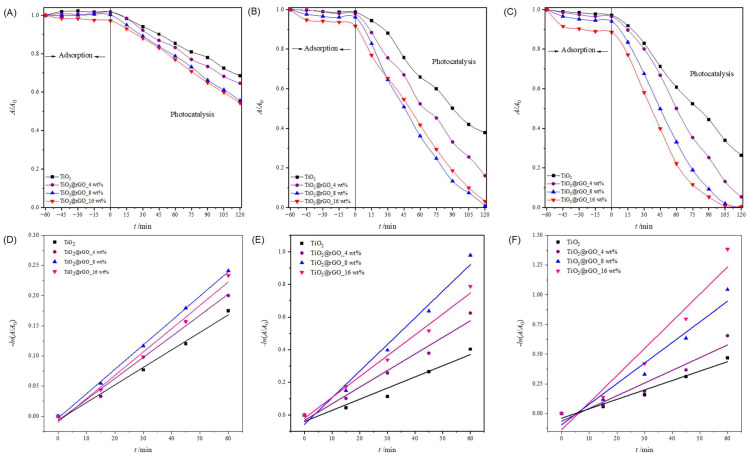
Change in relative absorbance of methylene blue from ultrapure water for (**A**) 0.1 g·L^−1^, (**B**) 0.5 g·L^−1^, and (**C**) 1 g·L^−1^ concentrations after using photocatalyst TiO_2_ and TiO_2_@GO nanocomposites. Change in relative absorbance of methylene blue as a function of photocatalytic degradation time using TiO_2_, TiO_2_@rGO_4 wt%, TiO_2_@rGO_8 wt%, and TiO_2_@rGO_16 wt% with simulated solar radiation for a catalyst concentration of (**D**) 0.1 g·L^−1^, (**E**) 0.5 g·L^−1^, and (**F**) 1 g·L^−1^.

From the obtained results (Figure 7A–C and Table S3), it can be seen that the adsorption of MB on the nanoparticles of the prepared catalysts increases with the increase in the amount of the catalyst. TiO_2_ nanoparticles showed the lowest adsorption of MB compared to the prepared TiO_2_@rGO nanocomposites. Comparing the prepared TiO_2_@rGO nanocomposites, it is evident that the amount of rGO in the nanocomposites promotes the improved adsorption of MB, which is expected because graphene-based materials have high affinity by aromatic compounds [54].

The linear dependence of the change in MB absorbance on the irradiation time is shown in Figure 7D–F. The values of the efficiency of photocatalytic degradation and the degradation rate constant, along with the corresponding values of the coefficient of determination (*R*^2^) of the MB dye degradation process for a catalyst concentration of 0.1, 0.5, and 1 g·L^−1^, are shown in Table S3. The values of the determination coefficient (*R*^2^) confirm that the decomposition rate follows a pseudo-first-order reaction.

It can be concluded that the effectiveness of the photocatalytic degradation of MB improves with the increase in the amount of catalyst from 0.1 to 1 g·L^−1^ for all prepared catalysts (Table S4). The number of active sites on the surface of the catalyst increases as the amount of catalyst available in the reaction medium increases, which consequently improves the effectiveness of the photocatalytic decomposition of MB. It can be seen that the catalyst mass concentration of 1 g·L^−1^ leads to a very small increase in photocatalytic degradation efficiency compared to a catalyst mass concentration of 0.5 g·L^−1^, while a catalyst mass concentration of 0.1 g·L^−1^ shows the lowest efficiency in the photocatalytic decomposition of MB. Due to the very small difference in the efficiency of the photocatalytic degradation of MB using catalyst mass concentrations of 0.5 and 1 g·L^−1^, the catalyst mass concentration of 0.5 g·L^−1^ was adopted as the optimal photocatalyst concentration for further photocatalytic studies.

#### 2.2.2. The Influence of the Initial Dye Concentration

**Figure 8:**
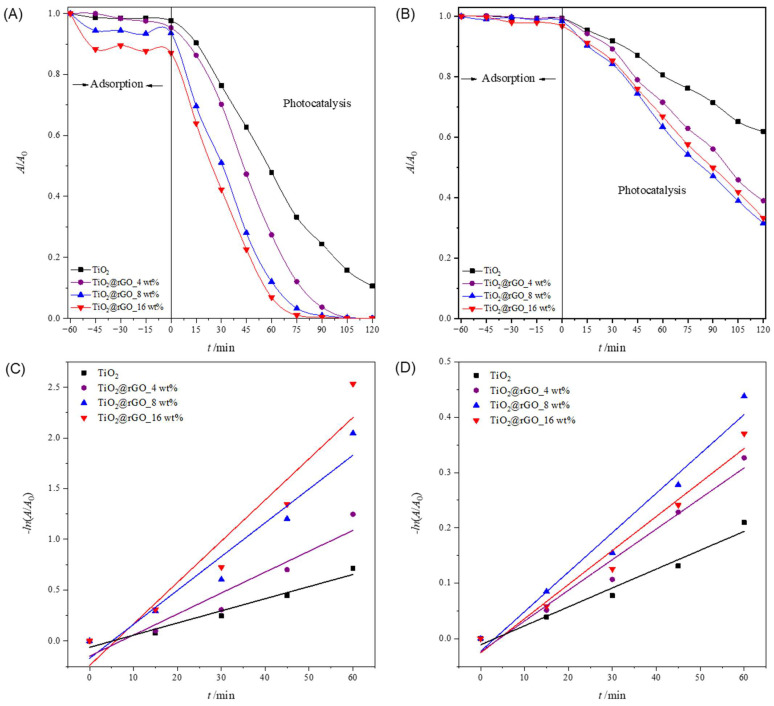
Change in relative absorbance of methylene blue under photocatalytic degradation using a concentration of 0.5 g·L^−1^ of the different photocatalysts (TiO_2_, TiO_2_@GO 4 wt%, TiO_2_@GO 8 wt%, and TiO_2_@GO 16 wt%). The initial concentration of MB in ultrapure water was (**A**) 5 mg·L^−1^ and (**B**) 15 mg·L^−1^. Change in the relative absorbance of methylene blue depending on the time of photocatalytic decomposition using TiO_2_, TiO_2_@rGO_4 wt%, TiO_2_@rGO_8 wt%, and TiO_2_@rGO_16 wt% with simulated solar radiation for the initial concentration of methylene blue pollutant of (**C**) 5 mg·L^−1^ and (**D**) 15 mg·L^−1^.

In order to examine the influence of the initial MB concentration on the effectiveness of the photocatalytic degradation performance of the TiO_2_@GO nanocomposites, different initial dye concentrations were prepared and tested. Three initial concentrations of MB, 5, 10, and 15 mg·L^−1^, were irradiated with simulated solar radiation, keeping other parameters constant (Figure 7B and Figure 8A,B).

It can be seen that the adsorption of MB on the nanoparticles of all prepared catalysts decreases with an increase in the initial concentration of the dye (Figure 8 and Table S5). The adsorption after 60 min for TiO_2_ nanoparticles is lower in contrast with the MB adsorption for TiO_2_@rGO nanocomposites. Moreover, the stronger adsorption properties are related to the amount of rGO in the materials. The highest adsorption was found for the TiO_2_@rGO_16 wt% sample, with the highest rGO content for both investigated initial concentrations. Comparing the adsorption values obtained for the TiO_2_@rGO nanocomposites, it is evident that the amount of rGO played an important role in the mechanism. However, it was noticed that the adsorption decreased with an increase in the initial dye concentration. This effect can be understood by the fact that the number of dye molecules increases but the amount of catalyst is constant, which consequently means that excess dye molecules cannot adsorb on the surface of the catalyst.

The linear dependence of the change in MB absorbance on the irradiation time is shown in Figure 7E and Figure 8C,D. The values of the efficiency of photocatalytic degradation and the degradation rate constants, along with the corresponding values of the coefficient of determination (*R*^2^) of the MB dye degradation process for the initial concentrations of 5, 10, and 15 mg·L^−1^, are shown in Table S6. The values of the coefficient of determination (*R*^2^) confirm that the decomposition rate follows a pseudo-first-order reaction.

The effectiveness of the photocatalytic degradation of MB decreases with increasing dye concentrations (Table S6). The higher initial dye concentrations slow down the photocatalytic reaction by shortening the path length of the photons entering the solution. Therefore, the production of charge carriers and reactive oxygen species was simultaneously reduced with an increase in the initial concentration of MB [55]. As a result, the concentration of reactive oxygen species produced at a higher initial dye concentration was not sufficient to efficiently promote the degradation of all the dye molecules in the water solution. For low dye concentrations, the number of reactive oxygen species was higher compared to the number of dye molecules. Comparatively, the complete removal of MB contaminants was achieved in a very short period for the lowest initial concentration compared to the higher initial dye concentration. As expected, the degradation of the dye was inversely proportional to the initial concentration of MB. It can be seen that under the current operating conditions, the lower the initial dye concentration, the better the removal efficiency. Based on the obtained results, 10 mg·L^−1^ was chosen as the optimal initial concentration of MB for an efficient photocatalytic process.

#### 2.2.3. Sunlight Photocatalytic Activity

**Figure 9:**
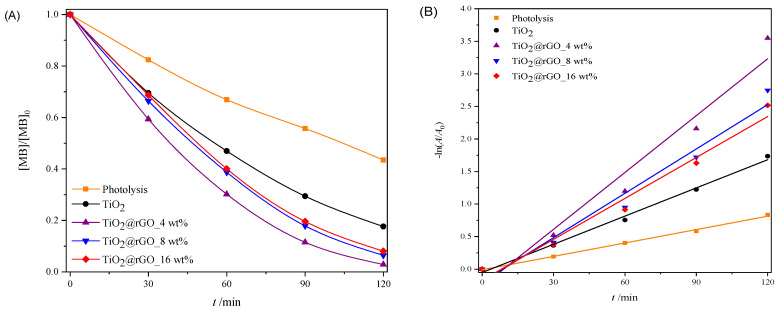
(**A**) Change in the relative absorbance of methylene blue (decolorization at 664 nm) depending on the time of photocatalytic degradation and (**B**) change in the relative absorbance of methylene blue depending on the time of photocatalytic decomposition during irradiation with natural sunlight radiation.

In order to test the ability of the used sunlight during the photocatalytic decomposition of MB, a photocatalytic performance assessment was carried out under natural solar radiation. The results of the effectiveness of the photocatalytic degradation of MB under natural solar radiation are shown in Figure 9. The efficiency of the photolytic decomposition of MB from ultrapure water without the presence of a photocatalyst was not negligible, with a degradation rate of 56.51% of the initial concentration after 120 min of exposure to natural solar radiation. As can be seen from Figure 9A, all prepared TiO_2_@rGO nanocomposites showed higher photocatalytic degradation than TiO_2_. The reason for the faster photocatalytic activity of the prepared TiO_2_@rGO nanocomposites compared to TiO_2_ is that rGO simultaneously acts as a charge separator and a photosensitizer [56]. Photocatalytic activity under natural solar radiation is closely related to the amount of rGO in the prepared nanocomposite. The fastest photocatalytic decomposition of MB was observed for the prepared TiO_2_@rGO_4 wt% nanocomposite. As the amount of rGO increased from 4 wt% to 16 wt% in the nanocomposites, the photocatalytic activity of TiO_2_@rGO nanocomposites gradually decreased. The increase in the amount of rGO limits photons in reaching the active sites on the surface of TiO_2_, which subsequently causes reduced photocatalytic activity under natural solar radiation. Another possibility is that graphene-based materials can act as a radical scavenger [57], causing, in this way, the inactivation of the radicals formed on the surfaces of TiO_2_ nanoparticles, constraining the photodegradation of MB. The prepared TiO_2_@rGO_4 wt% nanocomposite showed the best photocatalytic efficiency under the influence of natural solar radiation. The linear dependence of the change in absorbance of MB on the irradiation time is shown in Figure 9B. It can be seen that in terms of the effectiveness of the photolytic decomposition of MB, the source of visible radiation that is within the spectrum of the solar radiation source plays an important role. Photolytic degradation can be related to the amount of light absorbed by the MB molecules. Based on the absorbance spectrum of the MB, shown in Figure S2 it is visible that the absorbance maxima at 292 and 664 nm are attributed to π–π* and n–π* transitions, respectively [58]. As a result, MB molecules can be excited with photons, i.e., light sources with wavelengths that have radiation around the absorbance maxima. The consequence of the photolytic decrease in the absorbance of MB, i.e., the maximum at 664 nm when illuminated by natural solar radiation, is the result of the formation of reactive oxygen species and free radicals that are created from the visible part of the radiation of the solar radiation spectrum [58].

The values of the effectiveness of photocatalytic decomposition and the constant of the decomposition rate, with the corresponding values of the coefficient of determination (*R*^2^) of the process of decomposition of the MB dye under illumination with natural solar radiation, are shown in Table S7. The prepared TiO_2_@rGO 4 wt% nanocomposite had the highest degradation rate constant, which was about two times faster than that of the prepared TiO_2_ nanoparticles. The values of the coefficient of determination (*R*^2^) confirm that the decomposition rate follows a pseudo-first-order reaction.

#### 2.2.4. Influence of the Water Matrix

The degradation of pollutants by photocatalytic processes is greatly influenced by the composition of the water matrix that needs to be treated [59]. Natural waters are usually complex matrixes, containing diverse inorganic salts and organic substances. The composition of water has an influence on the kinetics of pollutant degradation. Four samples of natural water were chosen because of their importance for the environment and human health. The pH values of the water samples were between 7.16 and 8.37, which were slightly higher than that of the ultrapure water (pH = 6.77) that was used in the previous experiments. Ultrapure water has lower conductivity (0.378 µS cm^−1^) compared to natural inland waters (lake water = 351.2 µS cm^−1^ and river water = 275.8 µS cm^−1^) or water for human consumption (tap water = 489.0 µS cm^−1^), which have significantly higher conductivity, i.e., they contain many ions. However, seawater showed the highest conductivity, with 52,100 µS cm^−1^.

**Figure 10:**
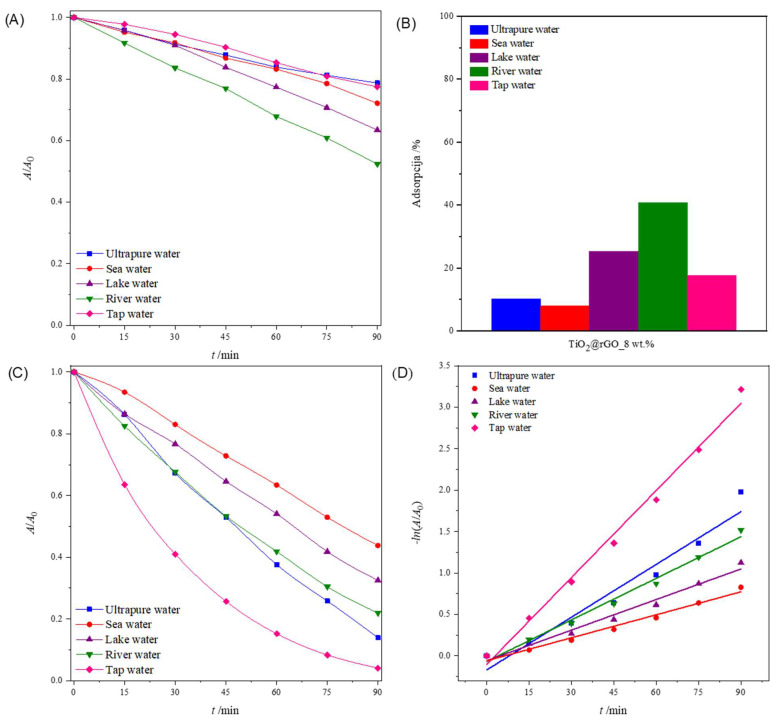
(**A**) Change in the relative absorbance of methylene blue (decolorization at 664 nm) depending on the time of photolytic degradation in different aqueous media; (**B**) total adsorption (ultrasound + stirring) of methylene blue after 75 min in different aqueous media for TiO_2_@rGO_8 wt% nanocomposite; (**C**) change in the relative absorbance of methylene blue (decolorization at 664 nm) as a function of photocatalytic degradation time in different aqueous media for TiO_2_@rGO_8 wt% with simulated solar radiation; and (**D**) change in the relative absorbance of methylene blue depending on the time of photocatalytic decomposition using TiO_2_@rGO_8 wt%.

Figure 10A shows the change in the relative absorbance of MB (decolorization at 664 nm) in different aqueous media irradiated with simulated sunlight. For 90 min, the greater photolytic decomposition of MB was observed in river and lake water compared to other water media. The consequence of the greater photolytic degradation of dyes in river and lake water is the presence of ions that act as reaction promoters and accelerate the photolytic degradation of MB. The values of the effectiveness of photolytic degradation and the constants of the degradation rate, along with the corresponding values of the determination coefficient (*R*^2^) of the MB dye decomposition process for different water media, are shown in Table S8. The values of the coefficient of determination (*R*^2^) confirm that the rate of photolytic degradation follows a pseudo-first-order reaction.

Figure 10B shows the total adsorption (ultrasound + stirring) of MB on the surface of the prepared TiO_2_@rGO_8 wt% nanocomposite. It can be seen that the prepared TiO_2_@rGO_8 wt% nanocomposite showed the significant adsorption of MB. The comparative study of the adsorption of MB on the surface of the photocatalyst for the different water media under study showed improved performance for river and lake waters, with 40.81% and 25.25%, respectively.

The results in Figure 10C show that the TiO_2_@rGO_8 wt% nanocomposite can efficiently degrade MB in all water matrices, including seawater, although additional time is required for complete degradation. The comparative efficiency in terms of the time required for degradation decreases in the order of tap water (95.98%) > ultrapure water (86.13%) > river water (78.07%) > lake water (67.48%) > seawater (56.22%), showing a significant dependence on the water medium used. This trend in the degradation activity of the TiO_2_@rGO_8 wt% nanocomposite seemed to be dependent on the complexity of the water matrices, as the natural matrices are more complex due to the exposure to different ions from different sources, which can compete with the active sites on the surfaces of photocatalytic nanocomposites. The values of the photolytic and photocatalytic degradation efficiency and the degradation rate constants, along with the corresponding values of the determination coefficient (*R*^2^) of the MB dye decomposition process for different water media, are shown in Table S8. The values of the coefficient of determination (*R*^2^) confirm that the decomposition rate follows a pseudo-first-order reaction (Figure 10D).

#### 2.2.5. Reuse of Photocatalysts

**Figure 11:**
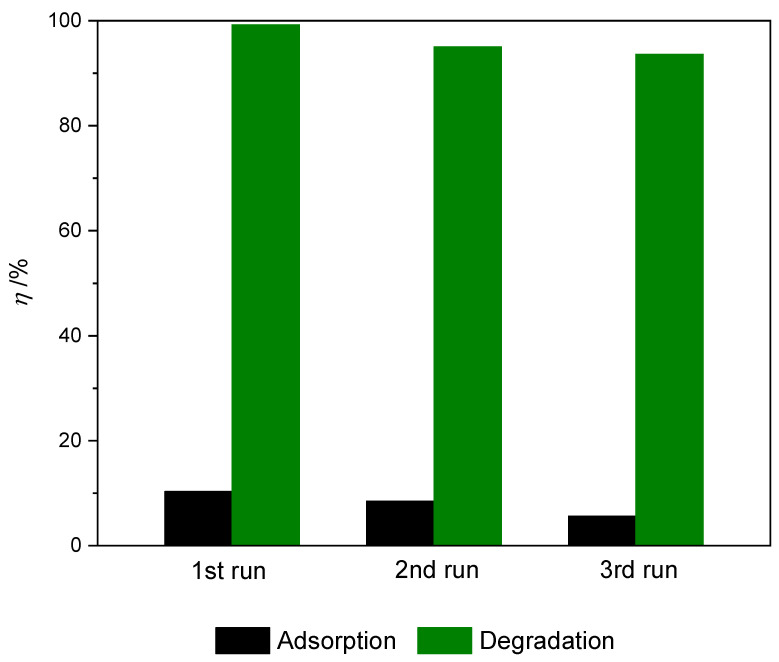
Dye removal during three consecutive cycles of adsorption and efficiency of photocatalytic degradation of methylene blue for prepared samples of TiO_2_@rGO_8 wt% nanocomposite during three consecutive cycles (*γ*_0_ = 10 mg·L^−1^, *V* = 30 mL, *m* (catalyst) = 15 mg, *T* = 22 ± 0.5 °C under simulated solar radiation).

For practical application, the recyclability and reusability of nanocomposite is a very important factor. Given that the prepared TiO_2_@rGO_8 wt% nanocomposite material showed superior photocatalytic activity when measuring different parameters of the photocatalytic process, it was selected as the most suitable nanocomposite for application testing in multiple consecutive cycles with the application of simulated solar radiation. The results of the conducted stability and reuse tests of the prepared TiO_2_@rGO_8 wt% nanocomposite are shown in Figure 11. After the first, second, and third cycles, the photocatalytic activity of the prepared photocatalyst decreased slightly, from 99.20% to 95.00% and 93.61%, respectively. The reduction in the effectiveness of photocatalytic activity was caused by the blocking of active sites on the photocatalyst surface due to the adsorption of MB molecules, even though the photocatalysts were washed with ultrapure water and ethanol between cycles [60]. Throughout the photocatalytic stability cycles, a slight change in the color of the photocatalyst was observed. The TiO_2_@rGO_8 wt% nanocomposite showed a gray-blue color after three cycles.

## 3. Materials and Methods

### 3.1. Chemicals

Commercially available flakes of graphite (particle size ≤ 50 μm) and titanium (IV) isopropoxide (Ti(C_3_H_5_O_12_)_4_, TTIP 97%) were acquired from Sigma-Aldrich (St. Louis, MO, USA), while nitric acid (HNO_3_, ≥65%) and sulfuric acid (H_2_SO_4_, 97%) were obtained from Honeywel Fluka (Seelze, Germany). Hydrochloric acid (HCl, 37%), potassium permanganate (KMnO_4_), sodium nitrate (NaNO_3_), hydrogen peroxide (H_2_O_2_, 30% *w*/*v*), i-propanol (C_3_H_7_OH), and acetylacetone (CH_3_(CO)CH_2_(CO)CH_3_) were supplied by Gram mol (Zagreb, Croatia). Methylene blue (MB), (7-(dimethylamino) phenothiazine 3-ylidene)-dimethyl azanium chloride, was purchased from Sigma-Aldrich (St. Louis, MO, USA). The solutions with the stated chemicals were prepared using ultrapure water. The ultrapure water was prepared in a LaboStar^®^ PRO water purification system (resistivity 18.2 MΩ/cm at 24.5 °C, 0.2 µm sterile filter, Siemens, Munich, Germany).

### 3.2. Preparation of TiO_2_@rGO

Firstly, graphene oxide (GO) was synthesized by the Hummers method previously reported [31]. The colloidal solution of TiO_2_ sol was prepared by dissolving titanium isopropoxide (TTIP) in 2-propanol (IPA) with constant stirring on a magnetic stirrer at a speed of 500 rpm. Then, acetylacetone (AcAc) was added and finally nitric acid (HNO_3_) was added. The molar ratio of the chemicals used to prepare the colloidal solution of TiO_2_ sol was as follows: TTIP:IPA:AcAc:HNO_3_ = 1:35:0.63:0.015 [61]. The prepared colloidal solution of TiO_2_ sol was bright yellow in color.

Samples of TiO_2_ nanoparticles and the TiO_2_@rGO nanocomposite with different weight percentages of synthesized GO (4, 8 and 16 wt%) were prepared by the hydrothermal/solvothermal method. Appropriate volumes of the prepared TiO_2_ sol colloidal solution and the synthesized GO suspension were mixed into the reaction mixture. To achieve homogenization, the reaction mixture was kept in an ultrasonic bath for 20 min, and then it was transferred to a Teflon container with a volume of 150 mL, which was then placed in a stainless-steel autoclave. The tightly closed autoclave was transferred to a laboratory oven and heated to 180 °C for 6 h. During this step, GO was transformed into reduced graphene oxide (rGO). After the end of the reaction time, the laboratory furnace was turned off, and the autoclave was cooled to room temperature overnight. The obtained synthesis product was separated from the reaction medium and washed 3 times with ethanol and then 3 times with deionized water and dried at 60 °C. The obtained powder of the TiO_2_@rGO nanocomposite was calcinated at 300 °C with a heating rate of 3 °C·min^−1^ for 2 h. The preparation of TiO_2_ nanoparticles was performed under identical conditions without the addition of GO and they were marked as TiO_2_. The synthesized nanocomposite materials were marked as TiO_2_@rGO_4 wt%, TiO_2_@rGO_8 wt%, or TiO_2_@rGO_16 wt%, where 4, 8, and 16 correspond to the amount of added GO. The reduction of the prepared GO was carried out by the hydrothermal method followed by thermal treatment in identical conditions to the as-prepared TiO_2_@rGO nanocomposites.

### 3.3. Characterization of TiO_2_@rGO Photocatalyst

The composition of the prepared materials was determined by X-ray powder diffraction (XRD) analysis, performed on a PANanalytical PRO MPD diffractometer with CuKα radiation (1.54056 Å) in the scan range of 8–80° (GO and rGO) and 10–80° in 0.033°/100 s increments.

Fourier transform infrared spectroscopy–attenuated total reflectance (FTIR-ATR) spectra of powder samples were recorded using a Shimadzu IR Spirit spectrometer with a diamond crystal in the measurement range of the wave number between 400 and 4000 cm^−1^, over 32 scans with a spectral resolution of 4 cm^−1^ at room temperature.

X-ray photoelectron spectroscopy (XPS) was used to determine the elemental composition and chemical structure of the elements detected on the surfaces of the prepared materials. The analysis was performed on powder samples of rGO, TiO_2_, and TiO_2_@rGO nanocomposite materials. Furthermore, the amount of titanium, carbon, and oxygen in the nanocomposite samples was determined. The analysis was performed on an XPS device (PHI-TFA, Physical Electronics Inc., Chanhassen, MN, USA) equipped with an Al monochromatic source that emitted photons at energies of 1486.6 eV. The analyzed area was 0.4 mm in diameter. High-energy-resolution spectra were obtained with the energy of the analyzer operating at a resolution of 0.6 eV and a transient energy of 29 eV. The accuracy of the binding energies was about ±0.3 eV. Quantification of the surface composition was derived from the XPS peak intensities, considering the relative sensitivity factors provided by the instrument manufacturer.

The morphology of the prepared TiO_2_ nanoparticles and TiO_2_@rGO nanocomposite was analyzed by transmission electron microscopy (TEM) on a JEM-2100 device (Jeol Ltd., Tokyo, Japan). The prepared powder samples were analyzed at a vacuum of <2.5 × 10^−5^ Pa and a voltage of 200 kV.

UV-Vis diffuse reflectance spectroscopy (UV-Vis DRS) was performed with the aim of determining the energy band of the prepared powder samples of TiO_2_ nanoparticles and TiO_2_@rGO nanocomposite materials. UV-Vis DRS measurements were performed on a UV-Vis-NIR spectrophotometer (UV-3600) equipped with an integrating sphere for reflection (ISR-3100 Integrating Sphere Attachment, 60 mm), Shimadzu, Kyoto, Japan. The measurement was carried out at room temperature, and barium sulfate (BaSO_4_) was used as a reference material.

The specific surface area was calculated by the BET method with a nitrogen adsorption analyzer (Quantachrome Nova 2000e, Anton Paar QuantaTec Inc., Graz, Austria).

### 3.4. Photocatalytic Experiments

The photocatalytic activity of pure TiO_2_ and TiO_2_@rGO nanocomposites with different proportions of rGO in the nanocomposite was tested in a quartz reactor with a volume of 50 mL, with constant stirring of the solution using a magenta instrument at 350 rpm. The MB dye was used as the modal solution (total volume = 30 mL) of the pollutant. Before testing the photocatalytic activity of the prepared materials, the photolytic activity of an MB solution was tested. Photolytic testing was carried out using the same process, but without adding the prepared photocatalyst to the reactor. Furthermore, the adsorption–desorption balance was examined, and the testing was carried out in the dark for 15 min in an ultrasonic bath due to the homogenization of the catalyst in the solution, and for 1 h by stirring on a magnetic stirrer. Then, the radiation was switched on and samples (aliquots) were collected in time intervals of 15 min over 120 min of illumination. Aliquots were centrifuged using a Minispin centrifuge (Eppendorf, Hamburg, Germany) for 10 min to separate the catalyst from the MB solution. The samples were placed in the quartz cuvette of a UV–Vis spectrophotometer (PerkinElmer, Hong Kong, China, Lambda 950 UV/VIS/NIR). The effectiveness of methylene blue dye degradation was monitored spectrophotometrically at 664 nm by measuring the absorbance. The samples were recorded in the wavelength range from 200 to 750 nm. The temperature of the reaction system was 22 ± 0.5 °C, and it was maintained using ventilation.

The photocatalytic decomposition of MB was followed by the application of a simulated solar lamp (Ultra Vitalux 300 W, Osram, Munich, Germany). As the distance of 0.20 m was applied during the experimental tests, the irradiation was expected to be approximately 5.3 ± 0.30 mW/cm^2^. The photocatalytic decomposition of the prepared materials was also monitored with the application of natural solar radiation, on July 30, 2021, in the period from 11 a.m. to 2 p.m. The intensity of UV-A and UV-B radiation was determined with a UV meter (model YK-35UV, Lutron Electronic, Coopersburg, PA, USA). The specifications of the used lamps are shown in Table 2.

In order to better simulate natural conditions, the photocatalytic decomposition of methylene blue was analyzed in different water media collected from a river, lake, and sea. River water was collected at the source of the Soča River in Slovenia. The water from the lake was collected on the glacial Lake Bled in Slovenia, and the seawater was collected on the Slovenian coast of the Adriatic Sea near the city of Piran. Degradation in tap water (Ljubljana, Slovenia) was also studied. 

The stability and photocatalytic efficiency of the photocatalyst was tested by experiments on the reuse of methylene blue decomposition from ultrapure water through three cycles. After each cycle of photocatalytic use, the photocatalysts were collected by centrifugation, washed with absolute ethanol and ultrapure water, and dried in an oven at 70 °C before reuse.

The photocatalytic degradation efficiency (*η*, %) of the prepared photocatalysts was calculated according to the following equation [62]:

(1) $$\eta =\frac{A_{0}-A_{t}}{A_{0}}\;\cdot 100\%$$

where *A*_0_ is the initial absorbance of the dye, and *A*_t_ is the absorbance of the dye at time t (min) during the photocatalytic process.

Pseudo-first-order kinetic models were used to investigate the kinetics of the photocatalytic degradation of dyes using the prepared nanocomposites. The linear form of the pseudo-first-order kinetic model is described by the following equation [62]:

(2) $$-ln\frac{A_{t}}{A_{0}}=k^{\prime }\cdot t$$

where *k*′ (min^−1^) is the rate constant of the pseudo-first-order reaction of the photocatalytic decomposition of dye, *A*_t_ is the absorbance of the dye after irradiation in time, t (min) during the photocatalytic process, and *A*_0_ is the initial absorbance of the dye.

## 4. Conclusions

This comprehensive work focused on the photocatalytic performance of TiO_2_@rGO nanocomposites synthesized using simple, environmentally friendly hydrothermal/solvothermal synthesis. The nanocomposites of TiO_2_@rGO exhibited adsorptive and photocatalytic activity under simulated solar light towards MB dye, a result of the formation of highly crystalline anatase nanoparticles finely dispersed over rGO nanosheets, which was confirmed by TEM analysis.

The prepared TiO_2_@rGO nanocomposite samples showed better photocatalytic activity than TiO_2_ nanoparticles for all investigated parameters of the photocatalytic process. In the case of the catalyst concentration, it was confirmed that with an increase in the amount of catalyst, the efficiency of MB removal under simulated solar radiation increased. The concentration of 0.5 g·L^−1^ proved to be optimal for all prepared photocatalysts, because, with the addition of a concentration of 1 g·L^−1^, the decomposition of methylene blue was significantly effective in relation to twice the amount of catalyst, i.e., the active surface of the catalyst. In the researched photocatalytic process, when the initial concentration of methylene blue was studied, it was shown that the concentration of pollutants significantly affected the rate of decomposition. As the initial concentration of methylene blue increased, the degradation efficiency decreased under simulated solar radiation. The initial concentration of 10 mg·L^−1^ of methylene blue proved to be the optimal concentration for pollutant removal for the prepared photocatalysts.

It has been proven that the nature of the water medium affects the photolytic and photocatalytic activity of MB decomposition. It was confirmed that the efficiency of MB degradation decreased in the order of tap water > ultrapure water > river water > lake water > seawater using a TiO_2_@rGO_8wt% photocatalyst under simulated solar illumination. The results of reuse showed that the prepared TiO_2_@rGO_8wt% nanocomposite can be used in three consecutive cycles while maintaining photocatalytic activity over 90%.

The best nanocomposite with TiO_2_ nanoparticles embedded on 8 wt% rGO sheets was found to show very high photocatalytic reactivity towards MB dye solution under simulated solar illumination due to π–π interactions between aromatic rings from rGO and MB molecules.

## Figures and Tables

**Table 1:** XPS survey of chemical elements in samples of interest.

Sample	C (at%) ± 0.3	O (at%) ± 0.3	Ti (at%) ± 0.3	O/Ti Ratio
rGO	85.5	14.5	/	/
TiO_2_	19.9	56.8	23.3	2.43
TiO_2_@rGO_4 wt%	20.9	55.3	23.8	2.32
TiO_2_@rGO_8 wt%	21.2	55.8	23.0	2.42
TiO_2_@rGO_16 wt%	24.3	53.7	22.0	2.44

**Table 2:** Specifications of used lamps.

Illumination Source	Power, W	*λ*, nm	Distance from Reactor, cm	UV-A and UV-B Intensity, mW·cm^−2^
Simulated solar radiation	300	280–780	20	5.3 ± 0.30
Natural sunlight irradiation	3.86 × 10^26^	100–1,000,000	/	6.5 ± 0.40

## Data Availability

The data of the study can be provided by the corresponding author upon reasonable request.

## Supplementary Materials

The following supporting information can be downloaded at: https://www.mdpi.com/article/10.3390/molecules28217326/s1.
