# Supplementary material for: Synergistic Remediation of Organic Dye by Titanium Dioxide/Reduced Graphene Oxide Nanocomposite
Source: Molecules. 2023 Oct 29;28(21):7326. doi: 10.3390/molecules28217326 (PMC10647384; doi:10.3390/molecules28217326)
Supplement: Supplementary file 1 [file molecules-28-07326-s001.zip › molecules-2654303-supplementary.pdf]

molecules

Article

Synergistic Remediation of Organic Dye by Titanium Dioxide/Reduced Graphene Oxide Nanocomposite

Martina Kocijan [1,*], Lidija Ćurković [1,*], Damjan Vengust [2], Tina Radošević [3], Vasyl Shvalya [4], Gil Gonçalves [5,6] and Matejka Podlogar [3]

1 Department of Materials, Faculty of Mechanical Engineering and Naval Architecture, University of Zagreb, Ivana Lučića 5, 10000 Zagreb, Croatia
2 Advanced Materials Department, Jožef Stefan Institute, Jamova Cesta 39, SI-1000 Ljubljana, Slovenia; damjan.vengust@ijs.si
3 Department for Nanostructured Materials, Jožef Stefan Institute, Jamova Cesta 39, SI-1000 Ljubljana, Slovenia; tina.radosevic@ijs.si (T.R.); matejka.podlogar@ijs.si (M.P.)
4 Department of Gaseous Electronics, Jožef Stefan Institute, SI-1000 Ljubljana, Slovenia; vasyl.shvalya@ijs.si
5 Centre for Mechanical Technology and Automation (TEMA), Mechanical Engineering Department, University of Aveiro, 3810-193 Aveiro, Portugal; ggoncalves@ua.pt
6 Intelligent Systems Associate Laboratory (LASI), 4800-058 Guimarães, Portugal
* Correspondence: martina.kocijan@fsb.hr (M.K.); lidija.curkovic@fsb.hr (L.Ć.)

Supplementary Materials

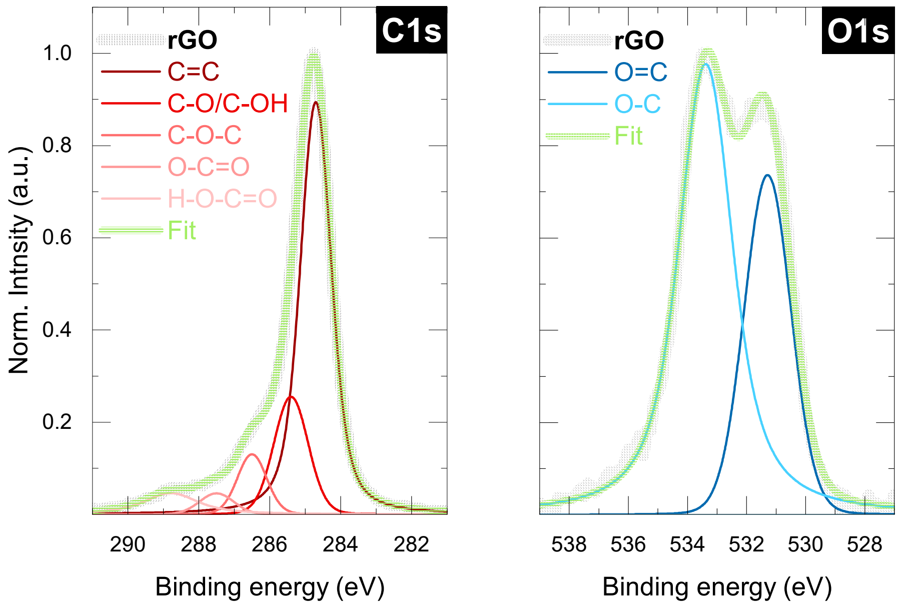

Figure S1. Deconvoluted C1s and O1s XPS spectra of rGO.

Table S1. Deconvoluted peak area for Ti2p and O1s peaks.

Sample	Area (Ti2p$_{3/2}$)	Area (Ti2p$_{1/2}$)	Area (O^{2-})	Area (OH)
rGO	/	/	/	/
TiO_2	1.44	0.58	1.49	0.28
TiO_2@rGO_4 wt%	1.41	0.59	1.48	0.29
TiO_2@rGO_8 wt%	1.42	0.59	1.49	0.29
TiO_2@rGO_16 wt%	1.41	0.59	1.50	0.28

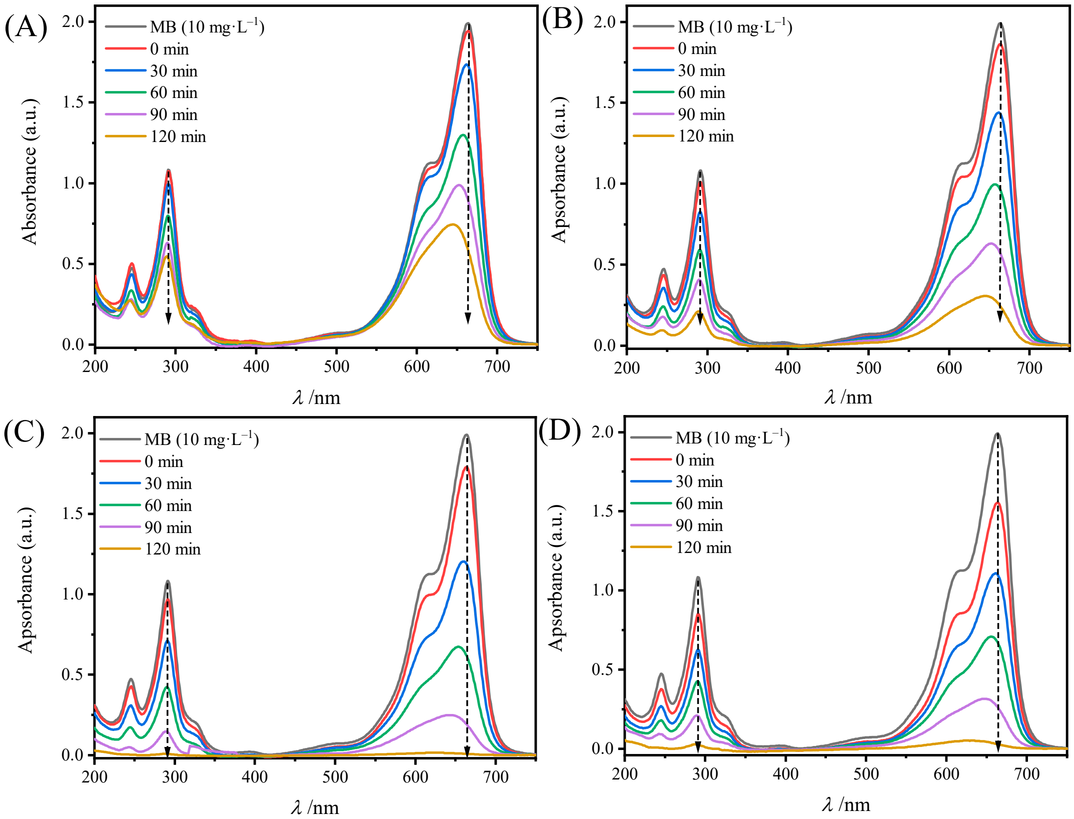

Figure S2. UV–Vis spectra of methylene blue dye decomposition in the time period of 0, 30, 60, 90 and 120 min under reaction conditions: γ_0(methylene blue)=10 mg·L^{-1}, V(solution)=30 mL, m(photocatalyst)=15 mg, T=22±0.5 °C, simulated Solar radiation, with the use of photocatalysts (A) TiO_2, (B) TiO_2@rGO_4 wt%, (C) TiO_2@rGO_8 wt% and (D) TiO_2@rGO_16 wt%.

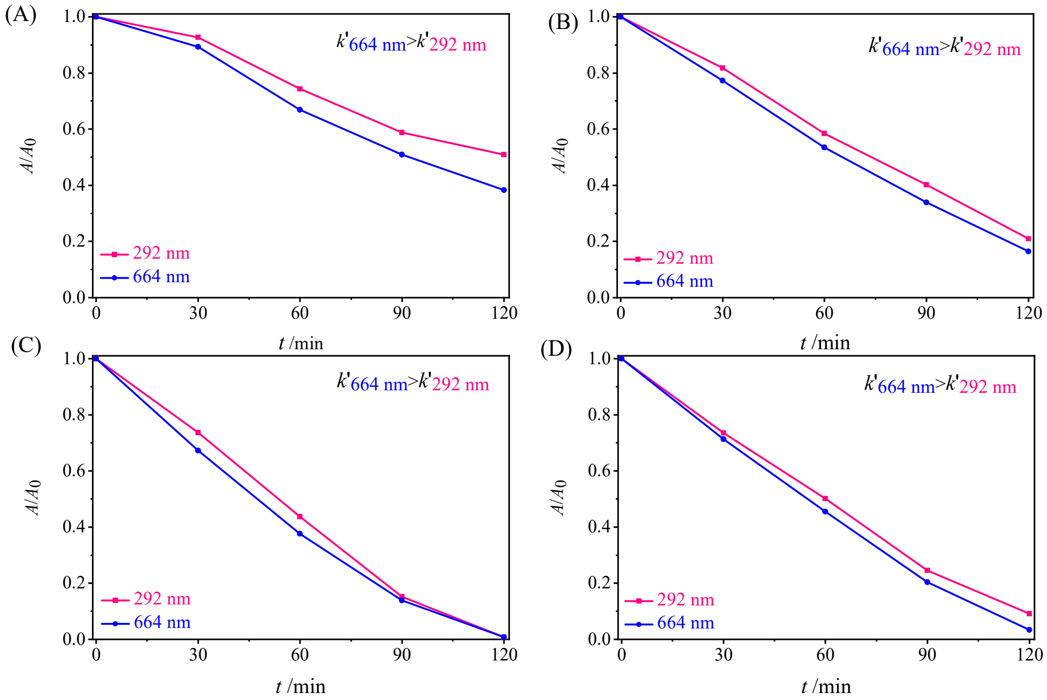

Figure S3. Change in the relative absorbance of methylene blue (aromaticity at 292 nm and decolourisation at 664 nm) depending on the time of photocatalytic degradation using (A) TiO_2, (B) TiO_2@rGO_4 wt%, (C) TiO_2@rGO_8 wt% and (D) TiO_2@rGO_16 wt% with simulated Solar radiation.

Table S2. Values of photocatalytic degradation efficiency (η), pseudo first-order reaction rate (k') and associated determination coefficient values (R^2) of the methylene blue dye decomposition process for a catalyst concentration of 0.5 g·L^{-1} at a temperature of 22±0.5 °C, with simulated Solar radiation.

**Sample**	η_{292} **(after 120 min), %**	k'_{292} **(after 90 min), × 10^{-3}, min^{-1}**	R^2	η_{664} **(after 120 min), %**	k'_{664} **(after 90 min), × 10^{-3}, min^{-1}**	R^2
TiO_2	49.14	6.06	0.95818	61.71	7.73	0.97021
TiO_2@rGO_4 wt%	79.05	10.23	0.98376	83.35	12.05	0.98486
TiO_2@rGO_8 wt%	99.20	20.55	0.92958	99.20	21.69	0.95805
TiO_2@rGO_16 wt%	90.83	15.30	0.96026	96.60	17.42	0.95955

Table S3. Achieved adsorption of methylene blue from ultrapure water during ultrasonic homogenization (15 min) and stirring (60 min), and total adsorption for 0.1, 0.5 and 1 g·L^{-1} after using photocatalyst TiO_2 and TiO_2@GO nanocomposites.

Sample	Adsorption (ultrasound-15 min), %	Adsorption (stirring-60 min), %	Total adsorption, %	Catalyst concentration, g·L^{-1}
TiO_2	0.39	/	0.39	0.1
TiO_2@rGO_4 wt%	1.70	/	1.70	
TiO_2@rGO_8 wt%	2.22	/	2.22	
TiO_2@rGO_16 wt%	5.45	2.82	8.22	
TiO_2	1.03	1.36	2.39	0.5
TiO_2@rGO_4 wt%	4.33	2.21	6.54	
TiO_2@rGO_8 wt%	6.37	3.93	10.30	
TiO_2@rGO_16 wt%	14.76	8.37	23.13	
TiO_2	1.41	2.48	3.89	1
TiO_2@rGO_4 wt%	11.01	3.53	11.54	
TiO_2@rGO_8 wt%	14.17	6.03	20.20	
TiO_2@rGO_16 wt%	34.31	11.48	45.79	

Table S4. Values of photocatalytic degradation efficiency (η), pseudo first-order reaction rate (k') and associated determination coefficient values (R^2) of the methylene blue dye degradation process for a catalyst concentration of 0.1, 0.5, and 1 g·L^{-1} at a temperature of 22±0.5 °C, with a simulated Solar lamp.

Sample	$\eta_{\text{(after 120 min)}}$, %	$k'_{\text{(after 60 min)}} \times 10^{-3}$, min^{-1}	R^2	Catalyst concentration, g·L^{-1}
TiO_2	33.52	2.91	0.99058	0.1
TiO_2@rGO_4 wt%	36.48	3.50	0.98754	
TiO_2@rGO_8 wt%	44.51	4.05	0.99920	
TiO_2@rGO_16 wt%	44.08	3.87	0.98641	
TiO_2	61.71	6.84	0.93168	0.5
TiO_2@rGO_4 wt%	83.35	10.18	0.96619	
TiO_2@rGO_8 wt%	99.20	16.30	0.97628	
TiO_2@rGO_16 wt%	96.60	12.78	0.98542	
TiO_2	72.86	7.95	0.95571	1
TiO_2@rGO_4 wt%	94.43	10.70	0.91211	
TiO_2@rGO_8 wt%	99.82	17.34	0.93935	
TiO_2@rGO_16 wt%	99.31	22.85	0.92421	

Table S5. Achieved adsorption of methylene blue from ultrapure water during ultrasonic homogenization (15 min) and stirring (60 min), and total adsorption for 5 and 15 mg·L^{-1} initial concentrations of methylene blue pollutant.

Sample	Adsorption (ultrasound-15 min), %	Adsorption (stirring-60 min), %	Total adsorption, %	Initial dye concentration, mg·L^{-1}
TiO_2	8.40	2.37	10.77	5
TiO_2@rGO_4 wt%	15.98	4.68	20.66	
TiO_2@rGO_8 wt%	19.59	6.47	26.06	
TiO_2@rGO_16 wt%	23.95	12.94	36.89	
TiO_2	0.09	0.59	0.68	15
TiO_2@rGO_4 wt%	0.32	0.77	1.09	
TiO_2@rGO_8 wt%	1.89	1.62	3.48	
TiO_2@rGO_16 wt%	5.79	3.33	9.12	

Table S6. Values of photocatalytic degradation efficiency (η), pseudo first-order reaction rate (k') and associated determination coefficient values (R^2) of methylene blue dye degradation process (γ_0=5 mg L^{-1}, V=30 mL, m(catalyst)=15 mg) at a temperature of 22±0.5 °C under simulated Solar lamp.

**Sample**	$\eta_{(after\ 120\ min)}$, **%**	$k'_{(after\ 60\ min)} \times 10^{-3}$, **min^{-1}**	R^2	Initial dye concentration, mg·L^{-1}
TiO_2	86.63	11.93	0.96318	5
TiO_2@rGO_4 wt%	99.82	20.62	0.91952	
TiO_2@rGO_8 wt%	99.91	33.35	0.94558	
TiO_2@rGO_16 wt%	99.92	40.71	0.92569	
TiO_2	37.70	3.41	0.96494	15
TiO_2@rGO_4 wt%	60.75	5.53	0.95784	
TiO_2@rGO_8 wt%	67.95	7.13	0.96225	
TiO_2@rGO_16 wt%	65.66	6.15	0.95977	

Table S7. Values of the efficiency of photolytic and photocatalytic degradation (η), pseudo first-order reaction rate (k') and the corresponding value of the coefficient of determination (R^2) of the methylene blue dye decomposition process (γ_0=10 mg L^{-1}, V=30 mL, m(catalyst)=15 mg) at a temperature of 22±0.5 °C with natural Sun radiation.

**Sample**	$\eta_{(after\ 120\ min)}$, %	$k'_{(after\ 120\ min)} \times 10^{-3}$, min^{-1}	R^2
Photolysis	56.51	6.86	0.99772
TiO_2	82.34	14.43	0.99474
TiO_2@rGO_4 wt%	97.12	29.10	0.96235
TiO_2@rGO_8 wt%	93.60	22.69	0.96743
TiO_2@rGO_16 wt%	91.92	20.95	0.97474

Table S8. Values of photocatalytic degradation efficiency (η), pseudo first order reaction rate (k') and associated determination coefficient values (R^2) of methylene blue dye degradation process for different water media, (γ_0=10 mg·L^{-1}, V=30 mL, m(catalyst)=15 mg at a temperature of 22±0.5 °C, with simulated Solar radiation.

Water matrix	$\eta_{(after\ 90\ min)}$, %	$k'_{(after\ 90\ min)} \times 10^{-3}$, min^{-1}	R^2
photolysis			
Ultrapure water	21.35	2.71	0.99415
Sea water	27.89	3.49	0.98291
Lake water	36.59	5.08	0.97657
River water	47.66	7.07	0.98819
Tap water	22.55	2.97	0.98257
photocatalysis			
Ultrapure water	86.13	21.24	0.94711
Sea water	56.22	9.25	0.97699
Lake water	67.48	12.31	0.97649
River water	78.07	16.73	0.98639
Tap water	95.98	35.01	0.98935
